# Morphine Exposure Reduces Nicotine-Induced Upregulation of Nicotinic Receptors and Decreases Volitional Nicotine Intake in a Mouse Model

**DOI:** 10.1093/ntr/ntac002

**Published:** 2022-01-06

**Authors:** Alicia J Avelar, Skylar Y Cooper, Thomas Douglas Wright, Sheavonnie K Wright, Montana R Richardson, Brandon J Henderson

**Affiliations:** Department of Biomedical Sciences, Joan C Edwards School of Medicine at Marshall University, Huntington, WV, USA; Department of Biomedical Sciences, Joan C Edwards School of Medicine at Marshall University, Huntington, WV, USA; Department of Biomedical Sciences, Joan C Edwards School of Medicine at Marshall University, Huntington, WV, USA; Department of Biomedical Sciences, Joan C Edwards School of Medicine at Marshall University, Huntington, WV, USA; Department of Biomedical Sciences, Joan C Edwards School of Medicine at Marshall University, Huntington, WV, USA; Department of Biomedical Sciences, Joan C Edwards School of Medicine at Marshall University, Huntington, WV, USA

## Abstract

**Introduction:**

Nicotine addiction remains a primary health concern as tobacco smoking remains the number one cause of preventable death in America. At the same time, America is still facing the threat of the opioid epidemic. While the prevalence of smoking combustible cigarettes or electronic nicotine delivery systems in the United States varies between 12% and 35%, the smoking rates among the opioid use dependent (OUD) population is 74%–97%. We examined changes in brain reward mechanisms in which co-use of nicotine and opioids may result in enhanced reward and reinforcement.

**Aims and Methods:**

Adult male and female α4-mCherryα6-GFP mice (C57BL/6J) were used in conditioned place preference (CPP) and microscopy assays to examine reward-related behavior and nicotinic acetylcholine receptor (nAChR) upregulation following treatments with saline, nicotine, morphine, or nicotine plus morphine. Following this, separate mice were trained in e-Vape self-administration assays to examine morphine’s impact on nicotine reinforcement.

**Results:**

We observed that nicotine and morphine coexposure in a CPP assay did not produce enhanced reward-related behavior when compared with nicotine or morphine alone. In parallel we observed coexposure reduced nicotine-induced upregulation of nAChRs on ventral tegmental area dopamine and GABA neurons. Additionally, we observed that concurrent morphine exposure reduced nicotine (plus menthol) vapor self-administration in male and female mice.

**Conclusions:**

While nicotine use is high among OUD individuals, our CPP assays suggest coexposure not only fails to enhance reward-related behavior but also reduces nicotine-induced changes in ventral tegmental area neurobiology. Our self-administration assays suggest that morphine exposure during nicotine acquisition reduces nicotine reinforcement-related behavior.

**Implications:**

While some may postulate that the co-use of opioids and nicotine may be driven by reward-related mechanisms, our data indicate that opioid exposure may hinder nicotine intake due to reduced upregulation of nAChRs critical for nicotine reward and reinforcement. Thus, the high co-use in OUD individuals may be a result of other mechanisms and this warrants further investigations into nicotine and opioid co-use.

## Introduction

Nicotine addiction remains a primary health concern. Despite decades of knowing the negative health consequences, tobacco use remains the number one cause of preventable death in America at ~480 000 each year.^1^ At the same time a number of Americans are opioid use dependent (OUD) and this has only worsened due to the COVID-19 pandemic. One feature that connects opioid use and nicotine is the high rates of smoking among those that use opioids. While the prevalence of smoking combustible cigarettes or electronic nicotine delivery systems (ENDS) in the United States varies between 12% and 35%, the smoking rates among the OUD population are 74%–97%.^2–5^ There are several reasons why co-use of nicotine and opioids may be high as using both may enhance subjective reward-related effects, increase pleasure with co-use, reduce withdrawal symptoms for each substance, or act as a substitution for the other substance.^6–8^ Additionally, both nicotine and opioids trigger disinhibition of dopamine neurons through direct actions on inhibitory GABA neurons.^9^ Accordingly, nicotine and opioids alter dopamine reward signaling in a similar manner and may potentiate each other.^9^ This highlights the *critical need* to understand the unique neurobiological consequences of opioid-nicotine co-use. It is possible that success rates for opioid cessation are so low because continued smoking or vaping facilitates neurobiological changes that contribute to addiction-related behavior. Therefore, it is necessary to determine how opioids may alter nicotine reward and reinforcement after long-term exposure.

We have previously shown that nicotine reward-related behavior correlates with the degree of α4-containing (α4*) and α4α6* nicotinic acetylcholine receptor (nAChR) upregulation.^10^ We have also previously observed that pharmacological agents that enhance nicotine reward-related behavior also enhance nAChR upregulation.^11^ Accordingly, we examined the impact of nicotine, morphine, and their combination on reward, reinforcement, and nAChR upregulation in the ventral tegmental area (VTA). While opioids used among OUD individuals vary, morphine is the prototypical opioid ligand. Furthermore, heroin which is abused commonly by OUD individuals exerts its effects by conversion to morphine. Thus, the examination of morphine’s actions will be useful in the context of gaining new insights related to heroin and nicotine coexposure.

## Methods

### Reagents

(−)-Nicotine dihydrogen ditartrate (product number—AC415660100) was obtained from ACROS Organics. Morphine sulfate salt pentahydrate was purchased from Sigma-Aldrich (St. Louis, MO). (−)-Menthol was obtained from Alfa Aesar (A10474). For e-liquids used in vapor self-administration, nicotine and menthol were mixed with propylene glycol and vegetable glycerin at a 50:50 ratio at a final concentration of 6 mg/mL (nicotine) and 15 mg/mL (menthol). This dose was selected based on our previous investigation that determined 6 mg/mL nicotine is preferred by mice in a dose–response assay and produces plasma cotinine levels consistent with human smokers.^12,13^ Regardless of presence of nicotine and/or menthol, the pH of e-liquids used in this study were 8.03 ± 0.04 and this falls in the normal range of e-liquid pH values (5.1–9.3).^14^ At this pH, we expect nicotine to be ~60% free nicotine (unprotonated).^14^ In all assays, nicotine content was based upon molecular weight of freebase. Doses of morphine in behavioral assays were based on previous literature.^13,15,16^

### Mice

All experiments were conducted in accordance with the guidelines for care and use of animals provided by the National Institutes of Health, and protocols were approved by the Institutional Animal Care and Use Committee at Marshall University. Mice were kept on a standard 12/12 light and dark cycle at 22°C and given food and water ad libitum. On postnatal day 21, mice were weaned and housed with same-sex littermates. Concomitantly, tail biopsies were taken and outsourced to a commercial laboratory for genotyping (Transnetyx, Cordova, TN). The construction of the α4-mCherryα6-GFP mice we used in these studies was previously described.^11^ The background for these mice are C57BL/6J and the mice were backcrossed to congenicity. These mice are available through the Mutant Mouse Resource & Research Center (MMRRC:68051-MU) for universal use. For microscopy assays, only mice that were transgenic for α6-GFP and homozygous for α4-mCherry were used (see below), with the exception of α6-GFP and α4-mCherry mice used for normalized Förster resonance energy transfer controls. All experiments used adult (3–5 months old) mice. Both male and female mice were used and numbers of each are detailed below in the methods for specific experiments.

### Conditioned Place Preference Assays

Conditioned Place Preference (CPP) assays were conducted in a three-chamber spatial place preference apparatus (Harvard Apparatus, PanLab) over a 10-day period, using male and female mice. Time spent in chambers was recorded by motion tracking software (SMART 3.0). The test consisted of three stages: pretest, injections, and post-test. An unbiased protocol was used where “drugs” (saline, nicotine [0.5 mg/kg], morphine [10 or 20 mg/kg], or morphine plus 0.5 mg/kg nicotine) were given immediately before confinement in the right white and gray chamber on “drug” days and saline was given immediately before confinement in the left white and black chamber on saline days. Each conditioning period lasted 20 minutes. Drug-naive mice that spent >65% of their time in one of the two conditioning chambers would have been removed from the study based on previously published methods^17–19^; however none exceeded this criteria and zero mice were excluded. Following this, mice were counterbalanced. During stage 2, intraperitoneal injections were given in the white and gray chamber (saline control, nicotine, morphine, or nicotine plus morphine, dissolved in saline) or white and black chamber (saline). The mice received their designated drug injections on days 2, 4, 6, and 8, and received saline injections on days 3, 5, 7, and 9. In the post-test stage, mice were again placed in the central chamber and given 20 minutes of free access to all chambers. Adult male and female mice, 3–5 months old, were used in CPP assays (54 males and 46 females total). Previous reports have shown that the use of α4-mCherryα6-GFP mice have displayed no differences in nicotine reward-related behavior as tested by CPP in comparison to C57BL/6J mice.^11,20^

Locomotor activity (distance traveled) during pre- and post-test recording sessions was not significantly different between the different drug treatment groups. Data are expressed as a change in baseline preference between the post- and pretest:


$$\begin{array}{l} {\left(drug_{paired}-\;saline_{paired}\right)}_{posttest}-{\left(drug_{paired}-saline_{paired}\right)}_{pretest} \end{array}$$

Experimenters were blind to drug treatments until all data analysis for the respective cohort were completed. To blind drug treatments, we had an individual, other than the experimenter, make saline and drug solutions and provide a nondescriptive label such as “solution A.” Once the CPP cohort was complete and mice were scored for the pre- and post-test, the experimenter was then informed the identity of the blinded solutions. This applied to only the drug-paired days, as the protocol dictates saline is given on saline-paired days. All drugs used in CPP assays were dissolved in saline at pH 7.4 and injected intraperitoneally.

### Confocal Imaging of Mouse Brain Slices

α4-mCherryα6-GFP mice were used in microscopy assays. Following the completion of CPP assays, mice were euthanized with CO_2_ and subjected to a swift cardiac perfusion with 10 mL ice-cold saline to reduce autofluorescence in the mCherry emission range. Brains were then promptly removed, flash frozen with acetone and dry ice, and then stored at −80°C. Brains were coronally sectioned (20 µm) using a cryostat, mounted with Vectashield (Vector labs, H-1000), and coverslipped. We targeted bregma −3.1 mm (anterior–posterior limits of −2.9 to −3.3 mm) because this region gave the most consistent sections that contained a large portion of the VTA, substantia nigra pars reticulata and substantia nigra pars compacta in a single slice.

A Leica SP5 TCSII confocal microscope was used to excite α6-GFP and α4-mCherry at 488 and 561 nm, respectively. ×20 images with a ×10 digital zoom were collected for the quantitative measurements of α4-mCherry and α6-GFP neuron raw integrated density. Normalized Förster resonance energy transfer was calculated using the PixFRET ImageJ plug-in to identify α4α6* nAChRs.

All experimenters were blind to drug treatment until all data analysis was completed. Approximately 30–60 VTA dopamine neurons and ≥30 VTA GABA neurons were imaged per brain slice for each mouse. Data from these images were averaged to provide raw integrated density values for each mouse. A total of 34 mice were used in confocal assays, aged 3–5 months (*n* provided in Figure 2).

### Self-administration

e-Vape self-administration (EVSA) was conducted in four air-tight chambers with interior dimensions of 21 cm L × 19 cm *W* × 12.5 cm *H* (La Jolla Alcohol Research, Inc [LJARI], La Jolla, CA).^12^ Two nosepokes with cue lights were mounted above the floor on the backside walls of the chamber. Airflow was vacuum controlled by an electric pump that allowed airflow at 1 L/min. U-well Crown IV atomizer tanks (0.40 ohms dual coil) were activated by a custom e-cigarette mod box (LJARI, La Jolla, CA). Vapor delivery settings were controlled by an e-Vape custom controller at 400°F and 65 W (LJARI, La Jolla, CA).

Mice were acclimated to vaporized deliveries using separate passive inhalation chambers that delivered 15 vapor deliveries of 6 mg/mL nicotine plus 15 mg/mL menthol for 5 daily sessions. This is a modification of a method previously used in our laboratory (three passive exposure sessions with propylene glycol and vegetable glycerin or nicotine-containing e-liquids),^12,21^ but results in a higher percentage of mice reaching acquisition criteria of 2:1 active:inactive nosepokes (~80% success vs. our previous ~50% success rate). Adult (3 months old), male (*n* = 20) and female (*n* = 21) mice began EVSA on a fixed-ratio (FR1) schedule on a Monday for 10 daily, 2-hour sessions, with a weekend break. Mice were singly housed in operant chambers.^12^ Nosepokes in the active hole of the operant chamber resulted in a 3-second delivery of vaporized e-liquids through the vapor entrance port. Inactive nosepokes were recorded with no consequences. Following a nosepoke and 3-second vapor delivery, a 30-second timeout period was initiated and signaled by a cue light in the nosepoke hole. Mice were trained on nicotine (salt) with 15 mg/mL menthol as this provides the most robust and consistent acquisition of self-administration behaviors.^12,21^ Following the 10-day FR1 protocol, mice continued on the same e-liquid but moved to a FR3 schedule for 5 days.

Given that mice were administered morphine on alternating days in the CPP paradigm, we replicated this in our self-administration paradigm. Accordingly, mice assigned to noncontingent morphine exposure during EVSA were given 10 mg/kg morphine (ip) on days 1, 3, 5, 7, 9, 11, and 13 of the 15-day EVSA protocol. Mice assigned to the control group were given saline injections (ip) on the same interval. To avoid simultaneous actions of nicotine and morphine, EVSA sessions ran from 8:30 to 10:30 am and morphine or saline injections were given at 3 pm.

### Statistical Analysis

All results are presented as mean ± SEM and all statistical analyses were performed using GraphPad Prism 9. CPP data were analyzed with a two-way ANOVA (sex × drug × interaction) with a post hoc Tukey for means comparison. Microscopy and self-administration data were analyzed by one-way ANOVA with a Tukey post hoc analysis. Power analyses (G*Power software, www.gpower.hhu.de) were used to determine efficient sample sizes (see Supplementary Material). Full statistical reporting for Supplementary Figures S1–S3 are provided in Supplementary Tables S1–S3. Outlier tests were conducted using a Grubb’s test (see Supplementary Material). Based on our outlier test, one mouse was excluded (see Supplementary Tables S4–S15).

## Results

### Reward-Related Behavior and Co-use of Nicotine With Morphine

We first examined the impact of coexposure to nicotine and morphine on reward-related behavior using adult mice in a CPP paradigm. Here, mice were assigned to cohorts including saline, 0.5 mg/kg nicotine, 10 mg/kg morphine, nicotine plus 10 mg/kg morphine, 20 mg/kg morphine, or nicotine plus 20 mg/kg morphine. Morphine doses were chosen due to prior morphine reward-related assays conducted in mice.^13,15,16,22^ Using a two-way ANOVA we did not detect a statistically significant sex difference in CPP (*F*_(1, 100)_ = 0.084, *p* = .77) but we detected a significant effect of drug treatment (*F*_(5, 100)_ = 10.5, *p* < .0001) and interaction (*F*_(5, 100)_ = 5.75, *p* = .0001) (see Supplementary Table S1 for full statistics). While we did not detect a sex difference in the overall two-way ANOVA, we observed sex-dependent responses to 10 mg/kg morphine (rewarding in males, not in females) and 20 mg/kg morphine (highly rewarding in females).

**Figure 1.**
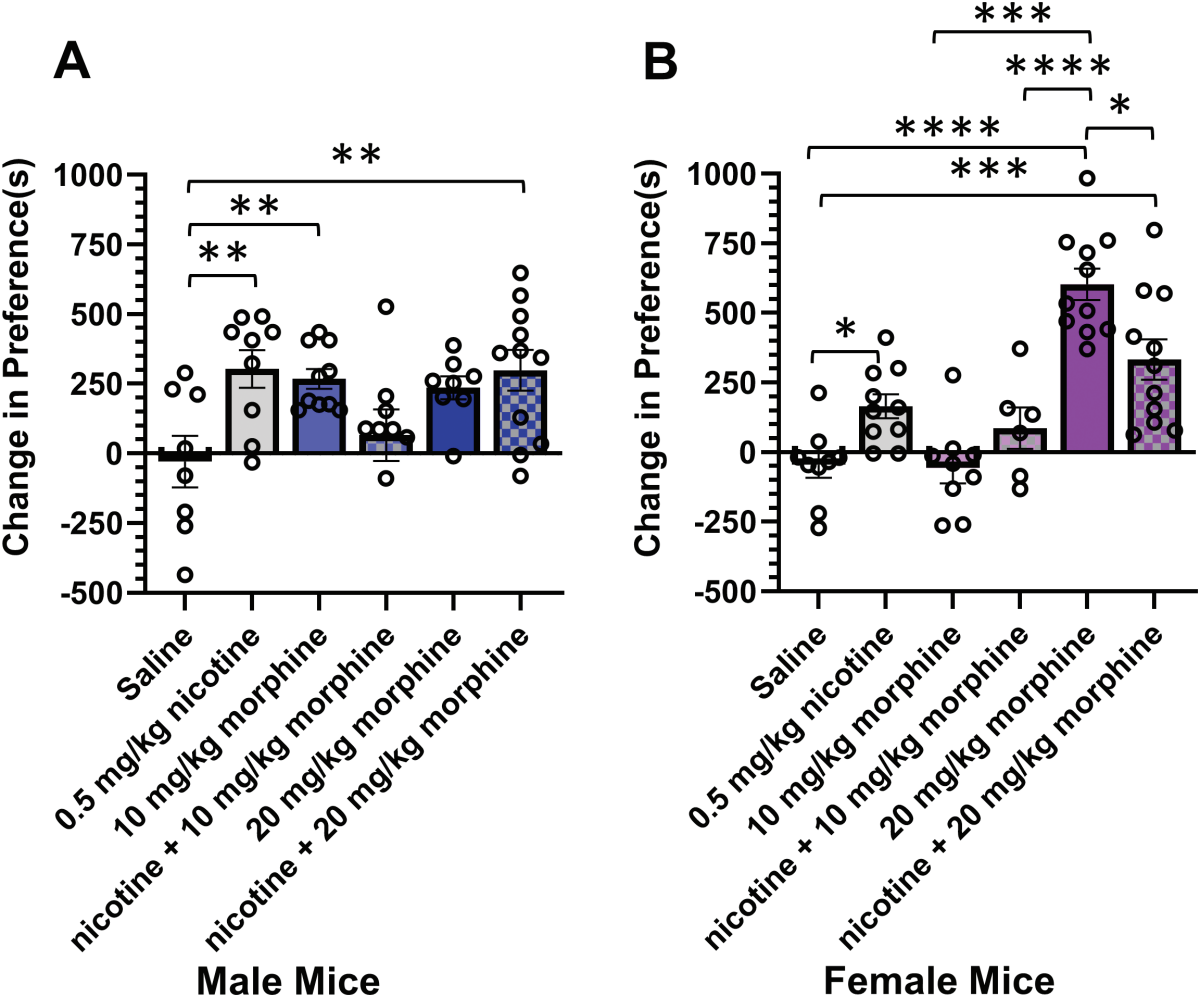
Reward-related behavior in a CPP assay. Male (A) and female (B) mice were used in a CPP assay to examine the reward-related behavior to saline, 0.5 mg/kg nicotine, 10 mg/kg morphine, 20 mg/kg morphine, and the combination of morphine plus nicotine. Data are mean ± SEM. Dots are data from individual mice (*n* = 7–11, per condition). *p < .05; **p < .01; ***p < .005; ****p < .001. CPP = conditioned place preference.

In male mice, we observed significant CPP to nicotine (*p* = .0098), 10 mg/kg morphine (*p* = .024), and nicotine plus 20 mg/kg morphine (*p* = .007) (Figure 1, A). Male mice exhibited similar CPP scores among nicotine alone, 10 mg/kg morphine, 20 mg/kg morphine, and 20 mg/kg morphine plus nicotine. We observed the combination of 0.5 mg/kg nicotine and 10 mg/kg morphine decreased reward-related behavior respective of either drug alone.

Similar to prior reports,^20^ we observed significant CPP to nicotine alone with female mice (*p* = .019). We did observe significant CPP to 20 mg/kg morphine (*p* < .0001) and 20 mg/kg morphine plus 0.5 mg/kg nicotine (*p* = .0006) (Figure 1, B). 20 mg/kg morphine alone exhibited a significant difference compared with nicotine alone (*p* < .0001).

In both males and females, the dose of morphine that produced significant CPP (10 and 20 mg/kg, respectively) exhibited reduced reward-related behavior when combined with nicotine. This suggests that the combination of nicotine and opioids, at rewarding doses does not produce enhanced pleasurable sensations.

### Impact of Nicotine and Morphine Coexposure on VTA nAChR Upregulation

**Figure 2.**
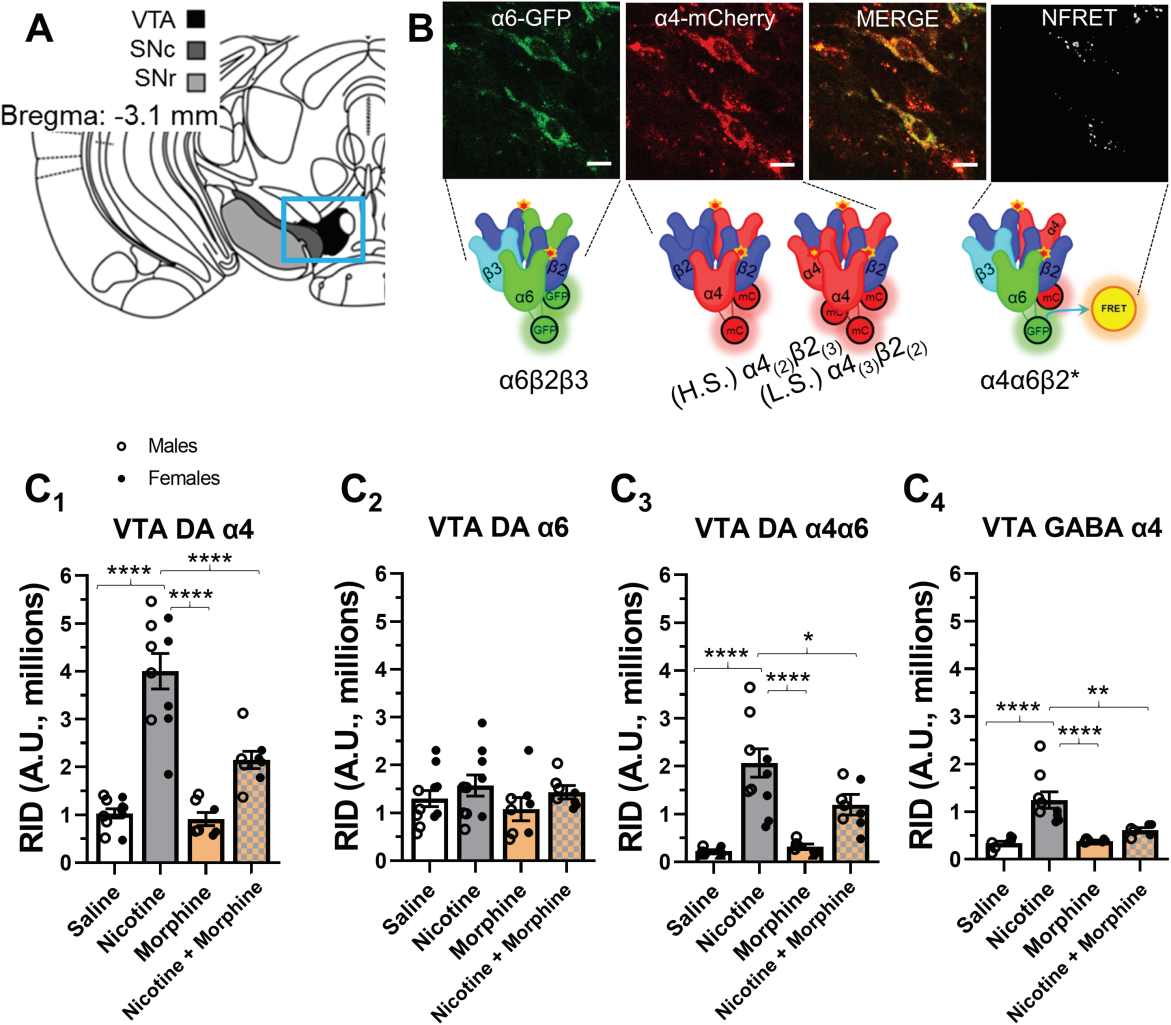
The impact of morphine treatment on nAChR upregulation. (A) Coronal schematic of region of interest. (B) Representative VTA neurons in α4-mCherryα6-GFP mice excited by 488 and 561 lasers and NFRET localization. (C_1–4_) Raw integrated density (RID) of fluorescent nAChRs in VTA dopamine and GABA neurons. Data are mean ± SEM. In (C), individual dots are data from individual mice (males are open circles, left; females are black circles, right). All groups, *n* = 6–10 per condition. *p < .05; **p < .01; ****p < .001. nAChR = nicotinic acetylcholine receptor; NFRET = normalized Förster resonance energy transfer; VTA = ventral tegmental area.

To examine changes in nAChR neurobiology, we used mice genetically modified to contain fluorescent α4-mCherry and α6-GFP nAChRs (α4-mCherryα6-GFP mice) that we have used previously to study nAChR upregulation.^10,11,20,23^ We have shown that reward-related behavior to nicotine may be critically dependent upon nAChR upregulation in VTA dopamine and GABA neurons.^10^ Accordingly, we focused our study on this brain area (Figure 2, A). In these studies, we detected no sex differences and both males and females exhibited the same trends (differing from CPP assays). Therefore, data are reported as sexes combined; however individual data for males and females are highlighted as white and black dots, respectively, in Figure 2 (see also Supplementary Figure S1). The α4-mCherryα6-GFP mice facilitate the study of α6*, α4*, and α4α6* nAChRs (Figure 2, B). Due to the fact that α6 nAChR subunits are selectively expressed in dopamine neurons within the VTA,^10,24^ the presence of α6-GFP was used to distinguish between dopamine and GABA neurons. To match CPP dosing, α4-mCherryα6-GFP mice were exposed to saline, nicotine, morphine (10 mg/kg), and nicotine plus morphine (10 mg/kg) using alternating daily injections of drug and saline to exactly match our CPP dosing paradigm. Following this, brains were extracted for analysis of nAChR fluorescence intensity (Figure 2, C_1–4_). For both VTA dopamine and GABA neurons, we detected a significant effect of drug treatment (Supplementary Table S2). As reported previously,^10,11,20^ CPP-consistent dosing of 0.5 mg/kg nicotine caused upregulation of α4* (*p* < .0001) and α4α6* (*p* < .0001) nAChRs on VTA dopamine neurons (and not α6* nAChRs) (Figure 2, C_1–3_). Similarly, nicotine caused a significant increase in α4* nAChRs on VTA GABA neurons (*p* < .0001) (Figure 2, C_4_). In all cases (VTA dopamine and VTA GABA neurons), 10 mg/kg morphine alone failed to cause any change in nAChR upregulation (Figure 2, C_1–4_). Interestingly, in all cases where nicotine-induced upregulation occurred, treatment with nicotine + morphine produced a reduction in nAChR upregulation compared with nicotine alone (VTA dopamine α4*, *p* = .0001; VTA dopamine α4α6*, *p* = .0177; VTA GABA α4*, *p* = .0028) (Figure 2, C).

### Impact of Noncontingent Morphine Exposure on Nicotine EVSA

**Figure 3.**
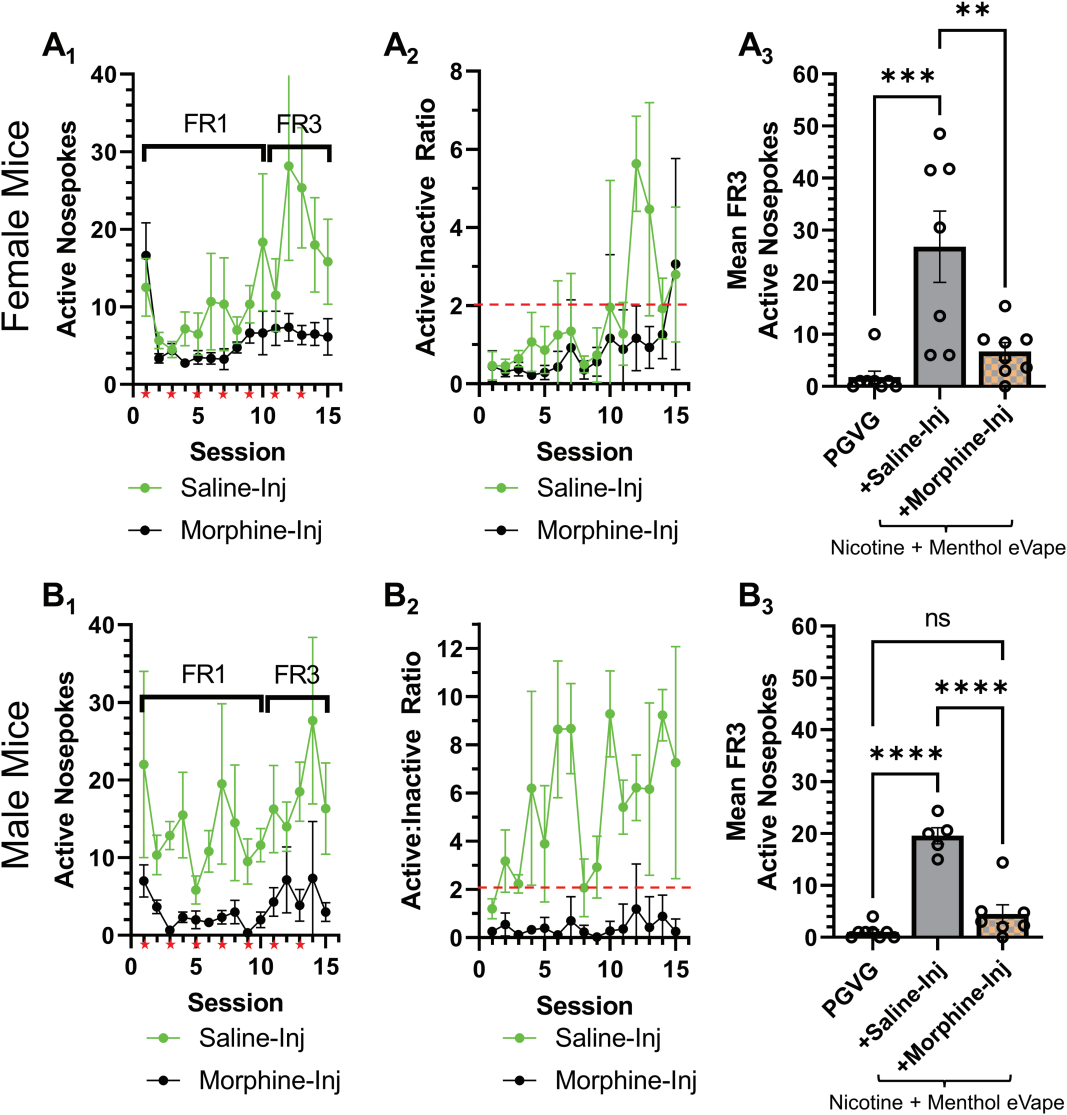
EVSA in mice assigned saline- or morphine treatment. (A_1_, B_1_) Active nosepokes for mice responding to 6 mg/mL nicotine (salt) plus 15 mg/mL (−)-Menthol with concurrent noncontingent saline- or morphine treatment. Stars indicate sessions with noncontingent morphine injections. (A_2_, B_2_) Active:inactive ratios of male and female mice during 15 session EVSA protocol (dotted line indicates threshold for 2:1 ratio). (A_3_, B_3_) Mean FR3 data for mice assigned PGVG, nicotine plus menthol with noncontingent saline injections, or nicotine plus menthol with noncontingent morphine injections. Data are mean ± SEM. Individual dots are data from individual mice (*n* = ≥7, per condition). **p < .01; ***p < .005; ****p < .001. For inactive nosepoke data, see Supplementary Figure S2. EVSA = e-Vape self-administration; PGVG = propylene glycol and vegetable glycerin.

Our microscopy data indicate that morphine exposure reduces nicotine-induced upregulation of nAChRs in brain regions and cell types key to nicotine reward and reinforcement. Due to the fact that nAChR upregulation is likely important for nicotine reward,^10^ we hypothesized that morphine-induced prevention of upregulation may decrease volitional nicotine intake. Therefore, we next used EVSA to examine how morphine exposure and a postulated reduction in nAChR upregulation impacted contingent nicotine intake. Our EVSA assays utilize the same technology used by human ENDS users and are therefore distinct from combustible cigarette and likely translational only to vaping-related behavior. Given that >90% of ENDS users use flavored e-liquids,^25–29^ we trained mice to self-administer vaporized nicotine (6 mg/mL) + menthol (15 mg/mL) and compared reinforcement-related behavior among two groups: (1) mice injected on alternating days with saline (7 injections total) during EVSA and (2) mice injected on alternating days with 10 mg/kg morphine (7 injections total) during EVSA (Figure 3).

Female mice triggered the active nosepoke to a similar degree regardless of their assignment to saline- or morphine-injected cohorts; however, female mice assigned to saline injections exhibited an increase in responding during the FR3 sessions while the morphine-injected females did not (Figure 3, A_1_). Similarly, saline-paired females discriminated between the active and inactive nosepokes (active:inactive ratio of ≥2) during FR3 sessions while morphine-injected females did not (Figure 3, A_2_). During both FR1 and FR3 schedules, saline-injected male mice exhibited more active nosepokes when compared with morphine-injected males (Figure 3, B_1_). Similarly, saline-injected male mice discriminated the active and inactive nosepokes while morphine-injected male mice did not (Figure 3, B_2_). Mean FR3 responding was analyzed between the morphine and saline cohorts and we found a significant effect of drug treatment (*F*_(2, 20)_ = 11.71, *p* = .0004; *F*_(2, 17)_ = 49.41, *p* < .0001, females and males, respectively, Figure 3, A_3_ and B_3_). We also observed a significant decrease in the number of active nosepokes between the saline- and morphine-assigned cohorts for both male and female mice (Figure 3, A_3_ and B_3_, *p* < .05, Supplementary Table S3). Together, these data show that morphine exposure during nicotine EVSA acquisition reduces nicotine intake.

## Discussion

Both nicotine and opioids have been characterized to mediate reward through mechanisms that can most simply be described as disinhibition of VTA dopamine neurons.^9,30^ Our CPP assays show a sex-specific effect of morphine in reward-related behavior. As investigations into male and female models expands, several investigations have revealed sex-dependent reward-related behavior for nicotine^17^ and even tobacco and ENDS flavorants.^20,23^ Here, we report that male mice exhibit reward-related behavior to 10 mg/kg morphine (and possibly 20 mg/kg, but not statistically significant) while female mice appear to exhibit no reward to 10 mg/kg morphine but are responsive to 20 mg/kg morphine. Our CPP assays also indicate that nicotine combined with rewarding doses of morphine (10 and 20 mg/kg for males and females, respectively) did not result in any enhancements of reward-related behavior but reduced reward-related behavior respective to morphine alone. As many drugs present an inverted-U for reward and then aversion, it is possible the combination of the two drugs has shifted the reward-response toward aversion. However, given the results of our male mice (decreased CPP with 10 mg/kg morphine + 0.5 mg/kg nicotine but then higher CPP with 20 mg/kg morphine plus 0.5 mg/kg nicotine) we believe impact on behavior and neurophysiology is likely more complex.

Our data also suggest that morphine exposure at a dose of 10 mg/kg (in both males and females) reduced nAChR upregulation of α4* nAChRs in both VTA GABA and dopamine neurons and α4α6* nAChRs in dopamine neurons. This result was interesting given that we noted a sex-specific effect on reward-related behavior but a nonsex-specific effect on nAChR upregulation. This suggests the dose of morphine necessary to impact nAChR upregulation may be lower (or much lower) than what is necessary for reward-related behavior. At this time, we have not tested further doses as these microscopy assays are extremely time intensive; however, our previous study into the relationship between nicotine reward-related behavior and nAChR upregulation^10^ shows the two events may be directly linked. Moreover, the nAChR subunits in the VTA that were prevented from upregulated are critical for nicotine reward.^10,31–34^ Based upon the finding that morphine decreases these key nAChRs from nicotine-induced upregulation, we hypothesized that concurrent morphine exposure during nicotine EVSA may decrease responding for nicotine. We did observe that mice injected with morphine on alternating days exhibited a significant decrease in vaporized nicotine intake. Therefore, the observation that morphine-injected mice that presumably exhibit reduced nAChR upregulation also exhibit reduced nicotine self-administration provides additional support for upregulation as a key mediator of nicotine reward and reinforcement. Given that our EVSA model translates to vaping-related behavior, we decided to include a flavor, given that most ENDS users prefer flavors.^35^ We have studied menthol most extensively and have shown that menthol enhances nicotine’s actions^11,12^ and only triggers “unique” neurobiological changes in the absence of nicotine.^36,37^ Therefore, of the chemical flavorants used with ENDS, menthol is the most thoroughly characterized and poses the lowest risk for three-way interactions with nicotine or morphine. Finally, our current success in training mice in EVSA assays are dependent on menthol for robust EVSA behavior.^12,21^

If morphine and nicotine are not working through a mechanism to enhance the reward of the other, why is it that there is such a high rate of nicotine use within OUD individuals? Both nicotine and opioids have severe withdrawal symptoms and this is a key issue when it comes to the low cessation rates of those wishing to abstain long term. It may be possible that one key factor to the dual use of nicotine and morphine may be that nicotine may reduce some of the somatic symptoms of opioid withdrawal or even the symptoms that occur during in-between bouts of opioid use. Despite this, the design of our assays likely place the timing of drug exposure to be during withdrawal states in mice. Nicotine and morphine have half-life values of ~7 and ~50 minutes, respectively, in mice.^38,39^ Accordingly, mice given morphine in PM noncontingent sessions have been nicotine free for ~5 hours. Mice placed in EVSA chambers in the AM were morphine free for ~13 hours. Thus, while they have not been placed in an extended abstinence paradigm they still likely exhibit nicotine and morphine withdrawal-related symptoms as both drugs produce withdrawal in early abstinence (in both humans and rodents).^40,41^ While this is a possibility, there are several assays directed toward withdrawal that will need to be completed and are clearly lacking in this current work.

One limitation of this study is our timeline of dual drug exposure being initiated at the same time. OUD individuals that are also nicotine dependent in many cases start as smokers and then transition to opioid use. Therefore, a necessary follow-up to this study is to examine the impact of dual use on nAChR upregulation and behavior using mice trained in nicotine EVSA assays and then exposed to an opioid. Here, we decided to use both drugs simultaneously as the first 10–14 days of nicotine exposure is critical for nAChR upregulation in rodent models.^38,42,43^ Accordingly, we wanted to determine how morphine + nicotine exposure during this timeline would impact nAChR upregulation.

In summary, this work provides novel insights into how opioid exposure may alter nAChR dynamics and then impact nicotine reward and reinforcement. Our data highlight that co-use of rewarding drugs may not depend on enhancements in rewarding sensations but may rely on other mechanisms.

## Supplementary Material

A Contributorship Form detailing each author’s specific involvement with this content, as well as any supplementary data, are available online at https://academic.oup.com/ntr.

ntac002_suppl_Supplementary_Data

ntac002_suppl_Supplementary_Taxonomy-form
