# Supplementary material for: Morphine Exposure Reduces Nicotine-Induced Upregulation of Nicotinic Receptors and Decreases Volitional Nicotine Intake in a Mouse Model
Source: Nicotine Tob Res. 2022 Jan 6;24(8):1161–8. doi: 10.1093/ntr/ntac002 (PMC9278828; doi:10.1093/ntr/ntac002)
Supplement: ntac002_suppl_Supplementary_Data [file ntac002_suppl_supplementary_data.docx]

**Supplemental Material**

| **Table S1. Statistical Reporting for Figure 1** | | | | | |
| --- | --- | --- | --- | --- | --- |
| **Statistical Test** | | | **Results** | | |
| Two-way ANOVA  Interaction | | | F_(5, 99)_ = 6.254  *p* < 0.0001 | | |
| Two-way ANOVA  Sex factor | | | F_(1, 99)_ = 0.063  *p* = 0.8021 | | |
| Two-way ANOVA  Drug factor | | | F_(5, 99)_ = 13.1  *p* < 0.0001 | | |
| **Male (Figure 1A)** | | | **Female (Figure 1B)** | | |
| **Statistical Test** | **Condition** | ***p* Value** | **Statistical Test** | **Condition** | ***p* Value** |
| *post hoc* Means Comparison | Saline vs. 0.5 mg/kg Nicotine | 0.0098 | *post hoc* Means Comparison | Saline vs. 0.5 mg/kg Nicotine | 0.01943 |
|  | Saline vs. 10 mg/kg Morphine | 0.024 |  | Saline vs. 10 mg/kg Morphine | >0.9999 |
|  | Saline vs. nicotine + 10 mg/kg Morphine | 0.9201 |  | Saline vs. nicotine + 10 mg/kg Morphine | 0.8006 |
|  | Saline vs. 20 mg/kg morphine | 0.0872 |  | Saline vs. 20 mg/kg morphine | <0.0001 |
|  | Saline vs. nicotine + 20 mg/kg Morphine | 0.007 |  | Saline vs. nicotine + 20 mg/kg Morphine | 0.0006 |
|  | 0.5 mg/kg nicotine vs. 10 mg/kg Morphine | 0.9987 |  | 0.5 mg/kg nicotine vs. 10 mg/kg Morphine | 0.1491 |
|  | 0.5 mg/kg nicotine vs. nicotine + 10 mg/kg Morphine | 0.1186 |  | 0.5 mg/kg nicotine vs. nicotine + 10 mg/kg Morphine | 0.9716 |
|  | 0.5 mg/kg nicotine vs. 20 mg/kg morphine | 0.9810 |  | 0.5 mg/kg nicotine vs. 20 mg/kg morphine | <0.0001 |
|  | 0.5 mg/kg nicotine vs. nicotine + 20 mg/kg Morphine | >0.9999 |  | 0.5 mg/kg nicotine vs. nicotine + 20 mg/kg Morphine | 0.3805 |
|  | 10 mg/kg morphine vs. nicotine + 10 mg/kg Morphine | 0.2348 |  | 10 mg/kg morphine vs. nicotine + 10 mg/kg Morphine | 0.7384 |
|  | 10 mg/kg morphine vs. 20 mg/kg morphine | 0.9994 |  | 10 mg/kg morphine vs. 20 mg/kg morphine | <0.0001 |
|  | 10 mg/kg morphine vs. nicotine + 20 mg/kg Morphine | 0.9992 |  | 10 mg/kg morphine vs. nicotine + 20 mg/kg Morphine | 0.0004 |
|  | Nicotine + 10 mg/kg morphine vs. 20 mg/kg morphine | 0.4871 |  | Nicotine + 10 mg/kg morphine vs. 20 mg/kg morphine | <0.0001 |
|  | nicotine + 10 mg/kg morphine vs. nicotine + 20 mg/kg morphine | 0.1007 |  | nicotine + 10 mg/kg morphine vs. nicotine + 20 mg/kg morphine | 0.1459 |
|  | 20 mg/kg morphine vs. nicotine + 20 mg/kg morphine | 0.9833 |  | 20 mg/kg morphine vs. nicotine + 20 mg/kg morphine | 0.0222 |

| **Table S2. Statistical Reporting for Figure 2** | | | | |
| --- | --- | --- | --- | --- |
| **Figure Panel** | | **Statistical Test** | **Conditions** | **Results** |
| Figure 2C_1_  VTA DA Neurons | | One Way ANOVA | VTA DA α4* nAChRs | F_(3, 32)_ = 40.95  *p* <0.0001 |
|  |  | *Post Hoc Means Comparison (Tukey)* | α4mC: saline vs. nicotine | *p <0.0001* |
|  |  |  | α4mC: saline vs. morphine | *p* = 0.9991 |
|  |  |  | α4mC: saline vs. Nicotine + morphine | *p* = 0.0098 |
|  |  |  | α4mC: nicotine vs. morphine | *p* < 0.0001 |
|  |  |  | α4mC: nicotine vs. nicotine + morphine | *p* = 0.0001 |
|  |  |  | α4mC: morphine vs. nicotine + morphine | *p* = 0.0193 |
| Figure 2C_2_  VTA DA Neurons | | One Way ANOVA | VTA DA α6* nAChRs | F_(3, 32)_ = 1.018  *p* = 0.3984 |
|  |  | *Post Hoc Means Comparison (Tukey)* | α6GFP: saline vs. nicotine | *p* = 0.7171 |
|  |  |  | α6GFP: saline vs. morphine | *p* = 0.8707 |
|  |  |  | α6GFP: saline vs. Nicotine + morphine | *p* = 0.9699 |
|  |  |  | α6GFP: nicotine vs. morphine | *p* = 0.3486 |
|  |  |  | α6GFP: nicotine vs. nicotine + morphine | *p* = 0.9677 |
|  |  |  | α6GFP: morphine vs. nicotine + morphine | *p* = 0.7107 |
| Figure 2C_3_  VTA DA Neurons | | One Way ANOVA | VTA DA α4α6* nAChRs | F_(3, 32)_ = 24.73  *p* < 0.0001 |
|  |  | *Post Hoc Means Comparison (Tukey)* | α4α6: saline vs. nicotine | *p <0.0001* |
|  |  |  | α4α6: saline vs. morphine | *p* = 0.9798 |
|  |  |  | α4α6: saline vs. Nicotine + morphine | *p* = 0.0050 |
|  |  |  | α4α6: nicotine vs. morphine | *p* < 0.0001 |
|  |  |  | α4α6: nicotine vs. nicotine + morphine | *p* = 0.0177 |
|  |  |  | α4α6: morphine vs. nicotine + morphine | *p* = 0.0256 |
| Figure 2C_4_  VTA GABA Neurons | | One Way ANOVA | VTA GABA α4* nAChRs | F_(3, 32)_ = 24.73  *p* < 0.0001 |
|  |  | *Post Hoc Means Comparison (Tukey)* | α4mC: saline vs. nicotine | *p <0.0001* |
|  |  |  | α4mC: saline vs. morphine | *p* = 0.9917 |
|  |  |  | α4mC: saline vs. Nicotine + morphine | *p* = 0.3251 |
|  |  |  | α4mC: nicotine vs. morphine | *p* < 0.0001 |
|  |  |  | α4mC: nicotine vs. nicotine + morphine | *p* = 0.0028 |
|  |  |  | α4mC: morphine vs. nicotine + morphine | *p* = 0.5549 |
|  |  |  |  |  |
|  |  |  |  |  |

| **Table S3. Statistical Reporting for Figure 3** | | | |
| --- | --- | --- | --- |
| **Figure Panel** | **Statistical Test** | **Conditions** | **Results** |
| Figure 3A_2_ | One Way ANOVA | Female EVSA | F_(2, 20)_ = 11.71  *p* = 0.0004 |
|  | Female FR3 EVSA  *Post Hoc Means Comparison (Tukey)* | PGVG vs. Nicotine | *p =* 0.0005 |
|  |  | PGVG vs. Nicotine + Morphine | *p* = 0.6175 |
|  |  | Nicotine vs. Nicotine + Morphine | *p* = 0.0039 |
| Figure 3B_2_ | One Way ANOVA | Male EVSA | F_(2, 17)_ = 49.41  *p* < 0.0001 |
|  | Male FR3 EVSA  *Post Hoc Means Comparison (Tukey)* | PGVG vs. Nicotine | *p <* 0.0001 |
|  |  | PGVG vs. Nicotine + Morphine | *p =* 0.1415 |
|  |  | Nicotine vs. Nicotine + Morphine | *p <* 0.0001 |
| *Note, all nicotine conditions were nicotine + menthol in EVSA assays | | | |

**Supplemental Figures**


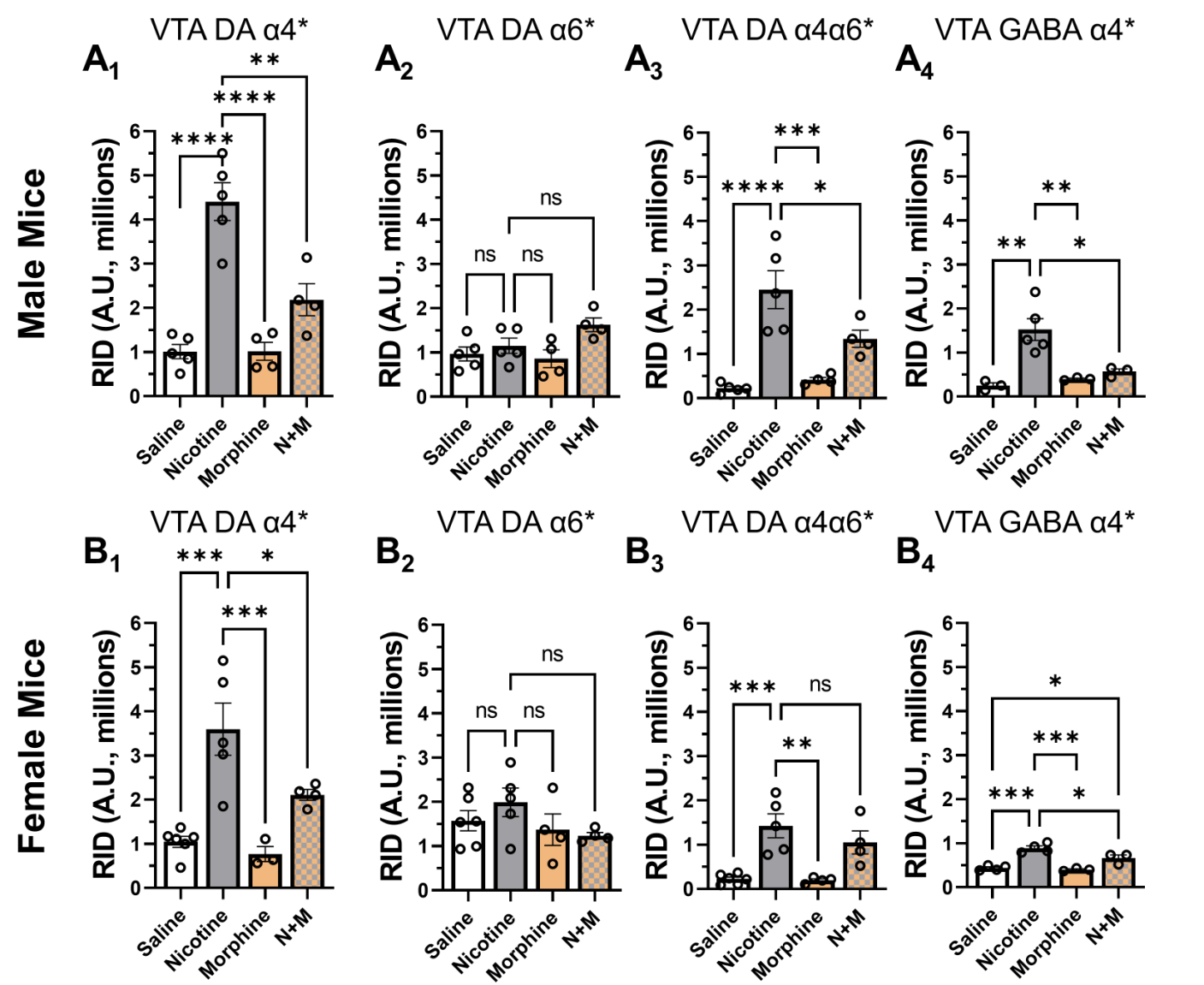


**Supplemental Figure 1.** nAChR subunit upregulation in dopamine and GABA neurons within the VTA separated by sex.

**
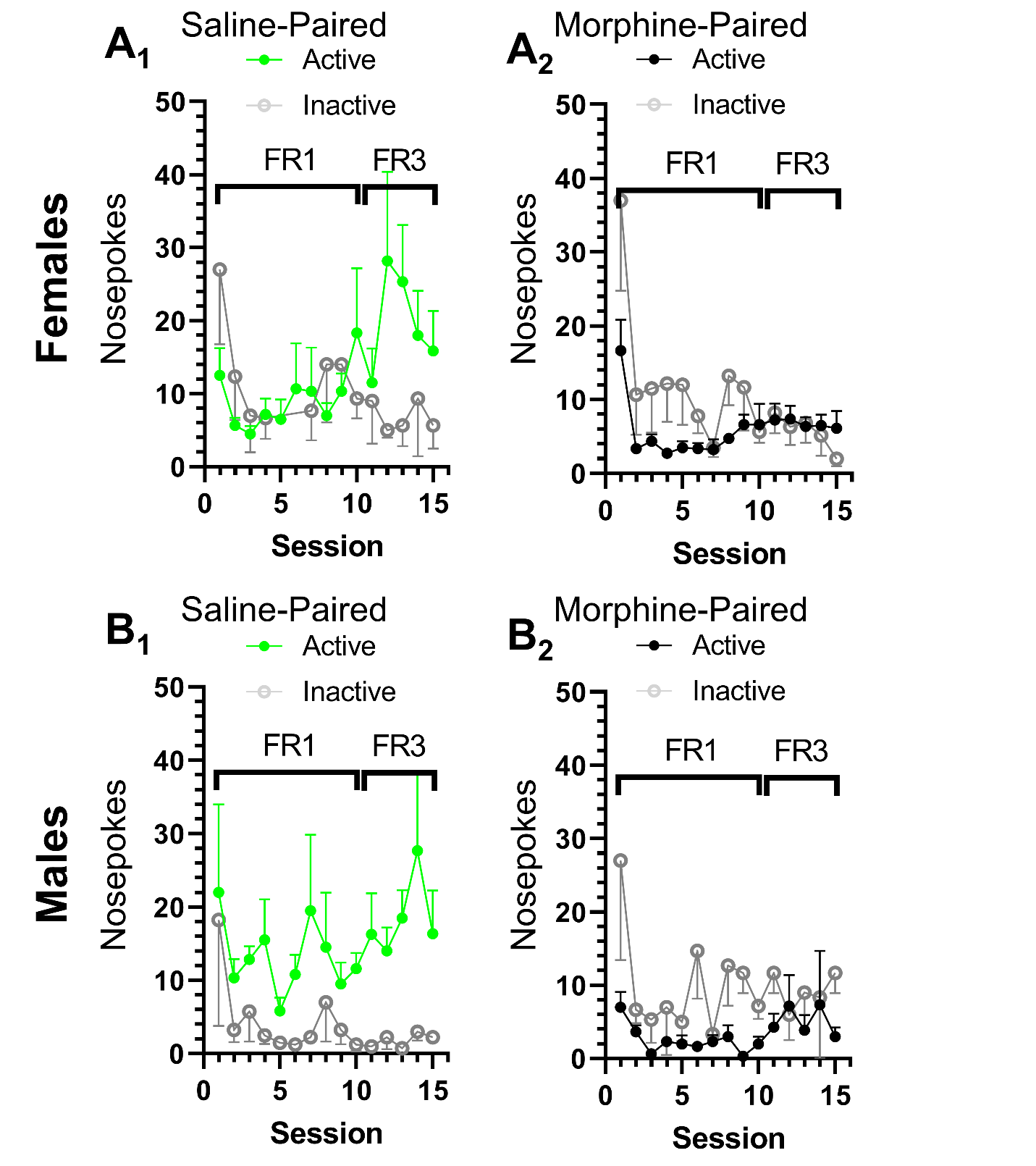
**

**Supplemental Figure 2.** (A_1-2_) Active and inactive nosepokes for male mice assigned to saline-injected and morphine-injected EVSA assays shown in the main paper Figure 3A_1_. The Green datapoints (A­­_1_) and black datapoints (A_2_) are the same data as presented in the main paper Figure 3A_1_. (B_1-2_) Active and inactive nosepokes for female mice assigned to saline-injected and morphine-injected EVSA assays shown in the main paper Figure 3B_1_. The Green datapoints (B­­_1_) and black datapoints (B_2_) are the same data as presented in the main paper Figure 3B_1_. These data highlight that mice may lose drug discrimination, or be less motivated to discriminate, when exposed to morphine.

Power Analyses

We performed several power analyses to determine the appropriate number of mice that will be required in this work using G*Power 3.1 (Franz Faul, University of Kiel, Germany). Our G*Power settings were: F tests (test family), ANOVA: fixed effects, one-way (statistical test), A priori (type of power analysis), effect size of 0.5-0.9, α error probability of 0.05, Power of 0.0.8, and number of groups varied depending on the assay.

e-Vape Self Administration

Number of groups: 3 (PGVG, saline-injected, and morphine-injected). Effect size = 0.7. Total sample size: 21 (8 mice per group). Critical F_(2, 21)_ = 3.467; actual power = 0.822. Considering both sexes, 48 mice will be necessary.

Conditioned Place Preference Assays:

Number of groups: 6 (Saline, 0.5 mg/kg nicotine, 10 mg/kg morphine, nicotine + 10 mg/kg morphine, 20 mg/kg morphine, and nicotine + 20 mg/kg morphine). Effect size = 0.6. Total sample size: 42 (7 mice per group). Critical F_(5, 36)_ = 2.477; actual power = 0.81. Considering both sexes, 84 mice will be necessary.

Confocal Microscopy:

Number of groups: 4 (Saline, 0.5 mg/kg nicotine, 10 mg/kg morphine, nicotine + morphine). Effect size = 0.9. Total sample size: 20 (5 mice per group). Critical F_(3, 16)_ = 3.239; actual power = 0.860.

Outlier Tests for Conditioned Place Preference (Female Mice)

| **Table S4. Outlier Test for Saline Cohort (Female mice, CPP)** | | | |
| --- | --- | --- | --- |
| **Row** | **Value** | **Z** | **Significant Outlier?** |
| 1 | -218.9300 | 1.236988 |  |
| 2 | -55.0000 | 0.066123 |  |
| 3 | -271.8000 | 1.614611 |  |
| 4 | 213.1700 | 1.849272 | Furthest from the rest, but not a significant outlier (P > 0.05). |
| 5 | -18.2233 | 0.196553 |  |
| 6 | 38.1367 | 0.599102 |  |
| 7 | -19.9133 | 0.184482 |  |
| 8 | -33.9400 | 0.084297 |  |
| 9 | -45.1800 | 0.004016 |  |
| Mean: -45.742  SD: 140.008  # of values: 9  Outlier detected? No  Significance level: 0.05 (two-sided)  Critical value of Z: 2.215 | | | |

| **Table S5. Outlier Test for 0.5 mg/kg nicotine (Female mice, CPP)** | | | |
| --- | --- | --- | --- |
| **Row** | **Value** | **Z** | **Significant Outlier?** |
| 1 | 160.31 | 0.0359 |  |
| 2 | 200.20 | 0.2563 |  |
| 3 | 74.38 | 0.6652 |  |
| 4 | 283.88 | 0.8691 |  |
| 5 | -4.81 | 1.2451 |  |
| 6 | 301.26 | 0.9964 |  |
| 7 | 412.06 | 1.8078 | Furthest from the rest, but not a significant outlier (P > 0.05). |
| 8 | 146.75 | 0.1352 |  |
| 9 | 81.99 | 0.6094 |  |
| 10 | -3.94 | 1.2387 |  |
| Mean: 165.2080  SD: 136.5489  # of values: 10  Outlier detected? No  Significance level: 0.05 (two-sided)  Critical value of Z: 2.2899 | | | |

| **Table S6. Outlier Test for 10 mg/kg morphine (Female mice, CPP)** | | | |
| --- | --- | --- | --- |
| **Row** | **Value** | **Z** | **Significant Outlier?** |
| 1 | -262.9900 | 1.266251 |  |
| 2 | -259.9400 | 1.247476 |  |
| 3 | 277.1900 | 2.058939 | Furthest from the rest, but not a significant outlier (P > 0.05). |
| 4 | -89.6200 | 0.199036 |  |
| 5 | -9.4900 | 0.294221 |  |
| 6 | -40.5000 | 0.103332 |  |
| 7 | -130.2500 | 0.449143 |  |
| 8 | -13.4775 | 0.269675 |  |
| 9 | 13.5000 | 0.435740 |  |
| Mean: -57.29  SD: 162.45  # of values: 9  Outlier detected? No  Significance level: 0.05 (two-sided)  Critical value of Z: 2.215 | | | |

| **Table S7. Outlier Test for 0.5 mg/kg nicotine + 10 mg/kg morphine (Female mice, CPP)** | | | |
| --- | --- | --- | --- |
| **Row** | **Value** | **Z** | **Significant Outlier?** |
| 1 | 69.75 | 0.3835 |  |
| 2 | 158.38 | 0.1485 |  |
| 3 | 135.38 | 0.2095 |  |
| 4 | -131.38 | 0.9168 |  |
| 5 | -85.06 | 0.7940 |  |
| 6 | 981.93 | 2.0352 | Significant outlier. P < 0.05 |
| 7 | 371.70 | 0.4171 |  |
| Mean: 214.39  SD: 377.14  # of values: 7  Outlier detected? Yes  Significance level: 0.05 (two-sided)  Critical value of Z: 2.02 | | | |

| **Table S8. Outlier Test for 20 mg/kg morphine (Female mice, CPP)** | | | |
| --- | --- | --- | --- |
| **Row** | **Value** | **Z** | **Significant Outlier?** |
| 1 | 984.87 | 2.0433 | Furthest from the rest, but not a significant outlier (P > 0.05). |
| 2 | 655.50 | 0.2823 |  |
| 3 | 717.01 | 0.6112 |  |
| 4 | 533.82 | 0.3683 |  |
| 5 | 371.00 | 1.2388 |  |
| 6 | 760.50 | 0.8437 |  |
| 7 | 754.69 | 0.8126 |  |
| 8 | 431.81 | 0.9137 |  |
| 9 | 508.32 | 0.5046 |  |
| 10 | 470.19 | 0.7085 |  |
| 11 | 442.00 | 0.8592 |  |
| Mean: 602.7009  SD: 187.0385  # of values: 11  Outlier detected? No  Significance level: 0.05 (two-sided)  Critical value of Z: 2.355 | | | |

| **Table S9. Outlier Test for 0.5 mg/kg nicotine + 20 mg/kg morphine (Female mice, CPP)** | | | |
| --- | --- | --- | --- |
| **Row** | **Value** | **Z** | **Significant Outlier?** |
| 1 | 155.12 | 0.7406 |  |
| 2 | 213.12 | 0.4994 |  |
| 3 | 310.37 | 0.0950 |  |
| 4 | 570.63 | 0.9871 |  |
| 5 | 798.13 | 1.9331 | Furthest from the rest, but not a significant outlier (P > 0.05). |
| 6 | 415.93 | 0.3439 |  |
| 7 | 580.00 | 1.0261 |  |
| 8 | 374.94 | 0.1734 |  |
| 9 | 106.99 | 0.9407 |  |
| 10 | 62.57 | 1.1254 |  |
| 11 | 77.69 | 1.0625 |  |
| Mean: 333.22  SD: 240.495  # of values: 11  Outlier detected? No  Significance level: 0.05 (two-sided)  Critical value of Z: 2.355 | | | |

Outlier Tests for Conditioned Place Preference (Male Mice)

| **Table S10. Outlier Test for Saline Cohort (Male mice, CPP)** | | | |
| --- | --- | --- | --- |
| **Row** | **Value** | **Z** | **Significant Outlier?** |
| 1 | 230.90 | 0.9902 |  |
| 2 | 17.57 | 0.1788 |  |
| 3 | 211.93 | 0.9181 |  |
| 4 | -260.13 | 0.8774 |  |
| 5 | 289.89 | 1.2146 |  |
| 6 | -79.93 | 0.1920 |  |
| 7 | -209.69 | 0.6856 |  |
| 8 | -436.10 | 1.5468 | Furthest from the rest, but not a significant outlier (P > 0.05). |
| Mean: -29.445  SD: 262.91  # of values: 8  Outlier detected? No  Significance level: 0.05 (two-sided)  Critical value of Z: 2.127 | | | |

| **Table S11. Outlier Test for 0.5 mg/kg Nicotine (Male mice, CPP)** | | | |
| --- | --- | --- | --- |
| **Row** | **Value** | **Z** | **Significant Outlier?** |
| 1 | -31.6700 | 1.657869 | Furthest from the rest, but not a significant outlier (P > 0.05). |
| 2 | 487.8800 | 0.912247 |  |
| 3 | 25.6900 | 1.374120 |  |
| 4 | 323.6900 | 0.100030 |  |
| 5 | 435.3700 | 0.652490 |  |
| 6 | 491.3800 | 0.929561 |  |
| 7 | 436.0575 | 0.655891 |  |
| 8 | 407.4300 | 0.514276 |  |
| 9 | 155.3925 | 0.732506 |  |
| Mean: 303.469  SD: 202.15  # of values: 9  Outlier detected? No  Significance level: 0.05 (two-sided)  Critical value of Z: 2.215 | | | |

| **Table S12. Outlier Test for 10 mg/kg Morphine (Male mice, CPP)** | | | |
| --- | --- | --- | --- |
| **Row** | **Value** | **Z** | **Significant Outlier?** |
| 1 | 189.7500 | 0.686210 |  |
| 2 | 295.9300 | 0.248080 |  |
| 3 | 175.8800 | 0.808254 |  |
| 4 | 176.9400 | 0.798927 |  |
| 5 | 277.1900 | 0.083185 |  |
| 6 | 436.0575 | 1.481079 | Furthest from the rest, but not a significant outlier (P > 0.05). |
| 7 | 407.4300 | 1.229182 |  |
| 8 | 155.3925 | 0.988526 |  |
| 9 | 407.4000 | 1.228918 |  |
| 10 | 155.3925 | 0.988526 |  |
| Mean: 267.736  SD: 113.648  # of values: 10  Outlier detected? No  Significance level: 0.05 (two-sided)  Critical value of Z: 2.29 | | | |

| **Table S13. Outlier Test for 0.5 mg/kg nicotine + 10 mg/kg Morphine (Male mice, CPP)** | | | |
| --- | --- | --- | --- |
| **Row** | **Value** | **Z** | **Significant Outlier?** |
| 1 | -89.63 | 0.5591 |  |
| 2 | 87.12 | 0.0776 |  |
| 3 | -526.87 | 2.1340 | Furthest from the rest, but not a significant outlier (P > 0.05). |
| 4 | 89.63 | 0.0866 |  |
| 5 | 87.12 | 0.0776 |  |
| 6 | 526.87 | 1.6616 |  |
| 7 | 205.56 | 0.5042 |  |
| 8 | 152.56 | 0.3133 |  |
| 9 | 57.87 | 0.0278 |  |
| Mean: 65.58  SD: 277.62  # of values: 10  Outlier detected? No  Significance level: 0.05 (two-sided)  Critical value of Z: 2.215 | | | |
|  | | | |

| **Table S14. Outlier Test for 20 mg/kg Morphine (Male mice, CPP)** | | | |
| --- | --- | --- | --- |
| **Row** | **Value** | **Z** | **Significant Outlier?** |
| 1 | -9.94 | 2.0972 | Furthest from the rest, but not a significant outlier (P > 0.05). |
| 2 | 260.00 | 0.2058 |  |
| 3 | 388.31 | 1.3005 |  |
| 4 | 202.18 | 0.2874 |  |
| 5 | 253.63 | 0.1515 |  |
| 6 | 277.44 | 0.3546 |  |
| 7 | 195.24 | 0.3467 |  |
| 8 | 320.12 | 0.7188 |  |
| Mean: 235.8725  SD: 117.212  # of values: 8  Outlier detected? No  Significance level: 0.05 (two-sided)  Critical value of Z: 2.127 | | | |

| **Table S15. Outlier Test for 10 mg/kg Nicotine + 20 mg/kg Morphine (Male mice, CPP)** | | | |
| --- | --- | --- | --- |
| **Row** | **Value** | **Z** | **Significant Outlier?** |
| 1 | 344.87 | 0.1896 |  |
| 2 | 648.06 | 1.4342 |  |
| 3 | 566.38 | 1.0989 |  |
| 4 | -5.81 | 1.2501 |  |
| 5 | 361.07 | 0.2561 |  |
| 6 | 34.88 | 1.0830 |  |
| 7 | 129.26 | 0.6956 |  |
| 8 | -80.62 | 1.5572 | Furthest from the rest, but not a significant outlier (P > 0.05). |
| 9 | 369.12 | 0.2891 |  |
| 10 | 425.88 | 0.5221 |  |
| 11 | 492.56 | 0.7959 |  |
| Mean: 298.6955  SD: 243.590  # of values: 11  Outlier detected? No  Significance level: 0.05 (two-sided)  Critical value of Z: 2.355 | | | |
